# Cigarette smoking after surviving breast cancer: A pilot study

**DOI:** 10.25082/ccr.2021.01.008

**Published:** 2021-11-01

**Authors:** Ban A. Majeed, Deepak Nag Ayyala, Steven S. Coughlin

**Affiliations:** 1 Department of Population Health Sciences, Medical College of Georgia, Augusta University, Augusta, GA, USA; 2 Institute of Public and Preventive Health, Augusta University, Augusta, GA, USA

**Keywords:** cigarette, former smoker, financial stress, income, addiction, breast cancer

## Abstract

**Background:**

Quitting smoking improves cancer survival and improves symptoms of cancer and its treatment. Cancer diagnosis presents a powerful motivation for leading a healthier lifestyle and embracing behavioral changes, such as quitting smoking. Many smokers quit after a cancer diagnosis, but some survivors continue to smoke. This study examined the characteristics associated with being a former rather than a current smoker among women treated for breast cancer.

**Methods:**

In this pilot, cross-sectional study, data were collected via postal surveys in women who had a history of smoking and breast cancer (N = 69). Descriptive and logistic regression analyses were conducted to identify factors associated with smoking status.

**Results:**

Of this sample, 13 were current smokers and 56 were former smokers. Age, race, education, and employment status were not associated with smoking status. Women with a higher income were significantly more likely to have successfully quit smoking (former smoking OR = 5.94, p < 0.05). Most women were light smokers and reported intentions to quit.

**Conclusion:**

The study attests to the addictive nature of smoking and the difficulty in achieving successful quitting even after breast cancer diagnosis. Results highlighted the role of low income as a barrier in smoking cessation. A follow up study is warranted to uncover potential barriers to smoking cessation in order to individualize tobacco treatment to meet the needs of motivated light smoking cancer patients. Intensive innovative tobacco treatment approaches are warranted, to reach successful cessation particularly among cancer patients with lower income.

## 1 Introduction

With increases in cancer survivors due to advances in cancer treatment and health care, quitting smoking among cancer survivors is necessary to improve long-term outcomes among both tobacco-related and other cancers. Although cigarette smoking is not an established, but a suggested, cause of breast cancer [1,2], continued smoking has detrimental effects on cancer treatment and survival, and symptoms associated with cancer and its therapy. For example, tobacco smoking after breast cancer diagnosis and treatment has been linked to increased overall and breast cancer-related mortality [3–5]. Although the health benefits of quitting are well-established, and cancer patients are strongly advised to quit, smoking sometimes persists even after a cancer diagnosis. Psychological factors such as anxiety, stress, depression, and fear of cancer recurrence have been shown to hinder cessation efforts in cancer patients [6].

Smoking is established and maintained by the intake of the dependence-producing drug, nicotine. Indeed, two thirds (68%) of adult smokers in the United States want to quit and half of them report a past-year quit attempt (stop smoking for > 24 hours) [7]. Unfortunately, the majority of quit attempts fail [8] largely because of nicotine dependence [9]. Some smokers may need up to 30 quit attempts before attaining successfully smoking cessation [8]. In patients with cancer, nicotine withdrawal symptoms [10], *e.g*. irritability, anger, anxiety, and insomnia, may exacerbate cancer associated physical, psychological, and financial stresses. The goal of this study was to examine the characteristics associated with being a former rather than a current smoker among women with a history of smoking and breast cancer.

## 2 Methods

### 2.1 Study sample

Data are from the Cardiovascular Disease outcomes among Breast Cancer Survivors (CVD-BCS), a cross-sectional study among a convenience sample of breast cancer survivors. Data were collected by postal survey questionnaires. Out of the 1000 eligible women invited to participate in the CVDBCS study, 165 of them responded (response rate 16.4%). Inclusion criteria included patients treated for breast cancer at Augusta University Health or the Georgia Cancer Center, they completed primary therapy for the disease, and they were over 18 years of age. All of the patients lived in the Central Savannah Regional Area; and had complete responses on questions pertaining to cigarette smoking status. Of 164 breast cancer survivors who responded to the survey, 156 women had complete data on cigarette smoking. The majority (n = 87, 55.8%) were never smokers. This study focuses on former and current smokers (N = 69).

### 2.2 Measures

#### 2.2.1 Cigarette smoking status

Women who have ever smoked a cigarette and currently smoke every day or some days were categorized as *current smokers*. Those who have ever smoked and responded *not at all* to the smoke now question were classified as *former smokers*. Furthermore, heaviness of smoking was assessed by number of cigarettes smoked per day (CPD). Information were also collected on past 12 months quit attempts and future intentions to quit.

#### 2.2.2 Participant characteristics

Demographic and other characteristics included in this study were age, race (White, and non-White), education (< high school or high school graduate, some college or college or advanced degree), annual household income (<$50,000 vs $50,000 +), number of people in household (1 vs 2 or more), employment status (employed vs not employed: retired, on disability, homemaker and temporary unemployed), marital status (married/with partner vs not married: single or widowed or separated/divorced), perceived general health (excellent/very good/good vs fair/ poor).

### 2.3 Statistical analysis

Descriptive and logistic regression analyses were conducted using R. We present point estimates in the form of percentages and odds rations as well as their 95% Confidence Intervals (CI). Significance level was set at *α* = 0.05. Overall and by participant characteristics, we computed the point estimates and 95% CI of the proportions of current and former smoking. Bivariate models of logistic regression were fitted where smoking status was the dependent variable (former smoker vs current smokers). Independent variables included characteristics such as age, race, education, and household income.

## 3 Results

Participant characteristics are presented in Table 1. Of breast cancer survivors with a history of tobacco use, 47 (68.12%) were aged 64+ years, 47 (73.44%) were non-white, 43 (66.15%) had at least some college education, and 21 (44.68%) had an annual income of $50,000 or higher. Of this sample, 13 were current smokers and 56 were former smokers. No variation in rates of smoking by age, race, education, employment, and household size were observed. Women with income of $50,000 or higher were significantly more likely to be former smokers than current smokers (former smokers 90.48% vs current smokers 9.52%; OR = 5.94, p = 0.04) as shown in Table 1.

Of the 13 current smokers, 10 women were light smokers (1–10 CPD). Only two (2/13) reported moderate – heavy smoking (11+ CPD). Many (7/13) of the current smokers reported at least one quit attempt in the past 12 months. Almost all (11/13) of the women reported intending to quit smoking in the future (one in the next 30 days, six in the next 6 months, one in the next year, and two later than 1 year).

## 4 Discussion

Continued smoking in breast cancer survivors, seen at an academic medical center in the southern United States, was observed in 23% of women with a history of cigarette smoking in this pilot study. Demographic factors, such as age, race, and education were not associated with smoking status. However, compared to former smoking, current smoking was positively associated with earning less than $50,000 per year. We posit that this relationship between lower annual household income and continued smoking after breast cancer treatment, could be explained by stress, anxiety, and depression – known predictors of relapse and continued smoking in the general population and in cancer patients [6, 11] – commonly induced by financial distress and the adverse impact of a cancer diagnosis on the financial well-being of patients [12]. In the study, the majority of current smokers wanted to quit and attempted to quit, yet relapsed to smoking. Failed attempts usually result from lack of support and underutilization of effective methods, combined counseling and pharmacotherapy [7]. The cost and unaffordability of approved cessation treatments, such as nicotine replacement therapy, Bupropion-SR, and Varenicline, may pose a barrier to use [13], especially among patients with lower income and cancer-related medical expenses, such as the case of current smokers earning less than $50,000 per year in this sample. This study highlights the need for intensive individualized tobacco treatment in breast cancer patients, utilizing effective and affordable modalities of counseling and medication.

The study is not free of limitations. Quitting date and use of cessation aid were not ascertained and thus it is unclear whether former smokers quit before or after breast cancer diagnosis and treatment. However, the available data were sufficient to achieve the overall goal of this study, which was to examine the characteristics of former smokers and compare them to breast cancer survivors who reported current smoking. A follow up study is warranted to uncover potential barriers to smoking cessation in order to individualize tobacco treatment to meet the needs of motivated light smoker cancer patients.

This pilot study revealed that, some women, albeit a small proportion, with a history of breast cancer treatment continue to smoke despite the health and financial benefits associated with smoking cessation. The findings also suggest that lower income is a risk factor for smoking among women with a history of breast cancer. Further investigation is important to shed light on potential barriers to successful cessation in breast cancer survivors and to identify ways to increase their cessation success rate.

## Figures and Tables

**Table 1:** Characteristics of study sample, overall and by smoking status (N = 69)

Characteristics	Overall	Current Smokers (n = 13)	Former Smokers (n = 56)			
	n (%)	n(%)	n(%)	OR	95% CI	*p*
Age						
< 64	22 (31.88)	5 (22.73)	17 (77.27)	Ref		
64+	47 (68.12)	8 (17.02)	39 (82.98)	1.43	0.39 – 4.96	0.57
Race						
White	17 (26.56)	11 (23.4)	36 (76.6)	Ref		
Non-White	47 (73.44)	2 (11.76)	15 (88.24)	2.29	0.53 – 15.97	0.32
Education						
≤ High School	22 (33.85)	7 (31.82)	15 (68.18)	Ref		
Some College +	43 (66.15)	6 (13.95)	37 (86.05)	2.88	0.38 – 10.37	0.10
Household income						
< $50,000	26 (55.32)	10 (38.46)	16 (61.54)	Ref		
$50,000 +	21 (44.68)	2 (9.52)	19 (90.48)	5.94*	1.32 – 42.46	0.04*
Number of people in household						
1	26 (38.24)	3 (11.54)	23 (88.46)	Ref		
2+	42 (61.76)	10 (23.81)	32 (76.19)	0.42	0.09 – 1.54	0.22
Employment status						
Not employed	58 (85.29)	9 (15.52)	49 (84.48)	Ref		
Employed	10 (14.71)	4 (40)	6 (60.00)	3.63	0.80 – 15.54	0.08
Marital status						
Married/partner	32 (47.06)	6 (18.75)	26 (81.25)	Ref		
Not married	36 (52.94)	7 (19.44)	29 (80.56)	0.96	0.28 – 3.24	0.94
Perceived general health						
Excellent-Good	55 (80.88)	9 (16.36)	46 (83.64)	Ref		
Fair-Poor	13 (19.12)	4 (30.77)	9 (69.23)	0.44	0.11 – 1.90	0.24

Notes:

* p <0.05
